# Globalization, migration health, and educational preparation for transnational medical encounters

**DOI:** 10.1186/1744-8603-2-2

**Published:** 2006-01-30

**Authors:** Peter H Koehn

**Affiliations:** 1 Professor of Political Science, University of MontanaMissoula, Montana, 59812, USA

## Abstract

Unprecedented migration, a core dimension of contemporary globalization, challenges population health. In a world of increasing human mobility, many health outcomes are shaped by transnational interactions among care providers and care recipients who meet in settings where nationality/ethnic match is not an option. This review article explores the value of transnational competence (TC) education as preparation for ethnically and socially discordant clinical encounters. The relevance of TC's five core skill domains (analytic, emotional, creative, communicative, and functional) for migration health and the medical-school curriculum is elaborated. A pedagogical approach that prepares for the transnational health-care consultation is presented, with a focus on clinical-clerkship learning experiences. Educational preparation for contemporary medical encounters needs to include a comprehensive set of patient-focused interpersonal skills, be adaptable to a wide variety of service users and global practice sites, and possess utility in addressing both the quality of patient care and socio-political constraints on migration health.

## Introduction

Migration, transmigration, [1] return migration, and remigration constitute defining elements of the current and future world order. More than 700 million people (including visitors on business or personal/family trips) traverse nation-state borders annually [2,3] and one million per week move between the global South and the global North [4]. The enormity of contemporary transnational mobility is illustrated by the case of Australia. In the past half century, Australia's "resident population has doubled, while the movement of people across its international boundaries (that is, into and out of Australia) has increased nearly one hundredfold" [5].

In a related development, cross-border migration for *settlement *in a new country increased more than twice as rapidly as the world's population grew during the last third of the Twentieth Century [6]. By 2000, about 185 million migrants resided legally or without documentation outside of their country of origin [2]. More than 55 percent of all residents of New York City, the world's most globally resettled metropolis, and 40 per cent of the residents of the state of Massachusetts are recent newcomers or children of immigrants/refugees [7,8].

Twenty-first Century demographic dynamics present new health-care challenges. In many German hospitals, for instance, migrant patients and their offspring occupy a majority of the beds in maternity and pediatrics wards. Since 2000, six of every ten babies delivered in New York City had at least one foreign-born parent [7]. Increasingly, hospitals across the United States are challenged to provide emergency care for undocumented migrants [9].

World-wide migration and the other interconnected transborder processes that constitute the heart of globalization [10-12] "are mixing people and microorganisms on an unprecedented scale" [[13], p.196, [12]] at breakneck speed [2]. As a consequence of the historic underfunding of research focused on tropical diseases, [15] globalization means that unprepared health centers and laboratory facilities in the North confront increasing exposure to neglected pathogens and health problems that afflict the South. People on the move can either "introduce new or previously eradicated diseases to the region of destination, or contract diseases unknown to the migrants' region of origin" [[16], p.85]. Recent examples include the rapid transcontinental transmission of Severe Acute Respiratory Syndrome (SARS), the spread of the polio virus from northern Nigeria to Indonesia [17-21], and the threat of an avian influenza pandemic [22]. Frequently, moving also adversely affects the migrant's mental well-being, adding to the burden of disease [16,11].

As more people in spatial transition compress the distance/time transmission of infectious and life-style-linked diseases, health protection, treatment, and promotion for migrants assume increasing consequence for individual patients, receiving societies and health-care systems, [23,24,2] and for global futures [25]. Downstream from many of the sources of infectious disease and the onset of chronic illness, [11] migrants and health professionals come together in medical-treatment and health-promotion encounters. In the context of contemporary population mobility, many health outcomes are shaped by transnational interactions among care providers and care recipients who meet in settings where nationality/ethnic match is not an option. In transnational consultations, clinicians and patients often deal with a wide variety of unfamiliar health threats and behaviors [26,27]. Prospects for reaching individually and socially positive outcomes are complicated when incongruent perspectives regarding physical and/or mental health problems, objectives, means for resolving problems, and outcomes prevail among professionals and service users of diverse nationality [28,29].

The rise of issues surrounding the movement of populations and pathogens across porous borders signals a growing concern with "migration health" [16]. Most studies of human migration and health emphasize national security concerns, surveillance, and/or policy responses involving population containment and exclusion. This review article focuses on the need to reorient a different dimension of global health governance: physician education. Education for transnational care merits special attention for at least three reasons: (1) medical professionals (physicians, mental-health-care providers, nurses, public-health specialists, and their teachers) form the backbone of the health sector throughout the world; (2) preparing physicians for cross-national medical encounters offers a change strategy that is proactive and encompasses the creation of health gain in a world characterized by continued population exchanges rather than a strategy that is exclusively or primarily reactive and preoccupied with eliminating disease; [30] and (3) medical-school education holds out the promise of contributing to the reduction of health disparities in an immediate and observable fashion.

## Physician education in a globalizing world

Throughout the global North and the global South, physicians are encountering patients in spatial transition from a multitude of dissimilar nation states [7] or ethnic communities. Contemporary medical-school curriculums and continuing education have not kept pace with the challenges that accompany an era of global mobility. In addition to multiple nationalities, physicians are challenged by bicultural, multicultural, and third-culture (different from both origin and host) [31] patients. Culture-competence education, initially intended for mastery of specific domestic two-culture interactions, [32] is of limited utility in today's diverse, hybrid, and rapidly changing patient-care environment [33,28]. Leyla Cinibulak reports, for instance, that "while health care providers [in the Netherlands] use a static notion of culture in their approach to migrant women, the Turkish migrant women's own approach towards the traditional values and taboos of their culture of origin and their religion is pragmatic and flexible" [[34], also [35]].

The multidimensional richness of human experience generates considerable intragroup variation [36,37]. Thus, migrants from a common sending place rarely share the same socio-economic and political backgrounds and mobility experiences [38,39]. Recipes of cultural characteristics miss the complexity of perspectives and behaviors that exist *within *ethnic groups due to varied social origins and behavioral inclinations, exposure to different experiences, mixed and emerging identities, and uneven transborder ties and involvements [40]. As Marjorie Kagawa-Singer and Shaheen Kassim-Lakha illustrate:

"What information does 'Chinese' convey? This man could have been born in Hong Kong, be a college professor who speaks five languages including English, and lives six months of the year in the United States and six months in Hong Kong. This man could also be a monolingual Chinese gentleman, born in the United States, unmarried, and living alone in Chinatown in New York, with little education and very poor [[33], p.579]."

Given the diverse, changing, and transgenerational nature of contemporary patient populations, [41] today's clinician must be skilled in identifying the special circumstances that surround and define each individual's health.

Educators increasingly appreciate that health is a global public good. The distribution of this good remains vastly unequal, however. Irrespective of ethnicity or culture, people who are poor "tend to experience more health problems in general over the course of their lives than do their more socioeconomically advantaged counterparts" [[42], p.504]. In large measure, disparities in health status reflect coping practices that are mediated by socio-economic position and ability to access and use health-care opportunities [28,43,12] In the global South, the most common social and economic determinants of medical problems and suffering are poverty, undernourishment, lack of access to safe water, absent or deficient sanitation, unhygienic housing conditions, [44,45] and, increasingly, a critical shortage of trained health workers – many of whom have emigrated to rich countries [46].

Individuals and families on the move frequently confront additional health risks associated with "health-compromising working and living conditions" along with inequities in health-care access and medical treatment [[47], p.126, [48,49]]. Individual health-care abilities and opportunities are not independent of forces linked to globalization – including economic, political, and military incursions that result in displacement and migration [50]. A recent African war-zone study carried out by Physicians for Human Rights concluded that the "first killer is flight" for desperately poor persons driven by conflict from a fragile existence into a hostile and personally threatening environment where health services are nonexistent or not functioning [51]. Migrants who leave behind safe social settings often are obliged to congregate in vulnerable spatial surroundings. Mobility simultaneously facilitates cumulative social-change processes (including isolation, marginalization, segregation, and discrimination) and risk-taking behaviors that are associated with increased susceptibility to and spread of noncommunicable as well as communicable disease [47,49,52-54]]. For this reason, HIV/AIDS researchers are devoting increased attention to the role of social disruption and migration to "hot spot" environments in fueling the epidemic [47,12,55]. To add insult to injury, the health problems of displaced and otherwise dislocated people tend to be officially "invisible" and are likely to be bypassed by potentially beneficial interventions [56].

"Irregular" and undocumented population movements pose special challenges of migration health. At all stages of migration (transportation, transit, and settlement), irregular migrants (including persons smuggled and trafficked) "are particularly exposed to contracting or transmitting diseases, to injuries or even death" [[16], pp.90–91, [57]]. The vulnerability of irregular migrants is exacerbated by poverty, powerlessness, the absence of social and legal protection, and lack of access to reliable health-care services. This situation often obliges them to seek medical attention through unofficial and unsafe means [16].

Although the reasons for disparities in health-care screening, medical procedures, morbidity, and mortality among persons who lack "voice" in biomedical institutions are multiple and complex, [58] the clinician/patient relationship constitutes an important contributing – and potentially mitigating – factor [59,60,48]. Carefully designed consultations enable public-health professionals to identify specific resources and support that will empower patients when addressing the challenges to positive health outcomes they face in the host society. Supportive actions on behalf of disadvantaged and underserved patients include facilitating access to social and health services provided by the host society; facilitating access to traditional healers and medicine as well as scarce (but, sometimes locally available) indigenous nutritional supplements; facilitating access to lay (community) health workers and intercultural mediators; assisting with the development of host-country language proficiency; promoting further education and credential (re-)certification; facilitating employment; help with moves into improved housing; [61] promoting the maintenance of children's healthy practices; [31,62] encouraging legal/policy coalition building with host-society institutions and transnational NGOs; and acting as the patient's advocate within the medical establishment and with government agencies and community associations.

In general, however, "the possibility of physicians working to improve contextual sources of distress" has been "overlooked" in medical education [[63], p.5, [64]]. Addressing power blinders [65] as well as social and political barriers to greater equity in access to health care falls outside the scope of most medical-school curriculums [64]. Without redirection, then, advocacy on behalf of a diverse and shifting circle of patients will continue to be viewed as peripheral, optional, and/or beyond one's capacity by future generations of physicians [66].

## Redirecting the medical-school curriculum: Preparing for patients in transition

In our age of globalization and dislocation, health-care initiatives and interactions need to be informed and supported by enhanced educational capacity [67]. The transnational competence (TC) framework [68] provides a valuable skill foundation for curriculum reform. The comprehensive set of practical skills that comprise the core of a TC education offer a promising emerging avenue for redirecting medical-school curriculums in ways that specifically and effectively address the connection between migration and health disparities. TC approaches transnational clinical encounters as micro-level interpersonal interactions that occur in a social/power context and are directly and indirectly shaped by macrolevel (global, regional, national, and local) structural factors. Advocacy is a conceptually integral skill component in TC preparation. Medical students are expected to address the social and power context, and to promote the health rights, of patients undergoing spatial, social, and identity transitions through specific recommendations that are critiqued, refined, and evaluated by faculty, preceptors, and care receivers.

TC education is based on a set of key principles, addresses the framework's five core skill domains, and utilizes a reinforcing pedagogical approach. Given that population mixing is widespread in the South as well as the North and that foreign-trained health-care professionals play a growing role in the health sector of many nations, [69,70] TC education needs to be available on a world-wide basis.

### TC principles

The first principle of TC education is patient-centered learning. The medical consultation is approached as a partnership, with the patient participating as teacher as well as learner and the student valuing the learning and mentoring dimensions of his/her role [58,71]. The patient's voice is treated as an indispensable source of expertise and experiential insight [37,72]. Rather than ignoring the perspectives of the least advantaged, [64] preparation for the TC encounter revolves around patient-oriented inquiries that are designed to promote congruent perspectives among care seekers and care providers on health status and health promotion – regardless of differences in national origin, ethnicity, cultural identity(ies), and socio-economic (and political) status. Findings from clinical studies consistently show that, when treated as an interactive, partnership-based process, [73] the medical consultation directly and indirectly improves the outcome of health-care interventions [74,59,58,71]. The TC approach anticipates, therefore, that health-care outcomes will be enhanced when patients also possess transnational competence [75] and demand and inspire corresponding skills on the part of the clinicians who consult with them.

TC's second core principle holds that patient advocacy is an indispensable physician activity. A TC education aims to move learners beyond patient sensitivity into responsiveness to patient needs. Across its five skill domains, the TC framework remains focused on two interconnected objectives: improved short- and long-term health outcomes for patients in spatial transition and reduced health inequities for dislocated populations and disadvantaged communities. Both objectives lie at the core of the People's Charter for Health that emerged from the People's Health Assembly held at Savar, Bangladesh, in 2000 [76].

A third TC principle centers on the resilience of underserved patients and families. TC preparation starts from the premise that patients in spatial transition are resilient and searches for ways to reinforce and expand their capacity to tap into potentially rich reservoirs of family, community, and transnational health-care resources.

### TC domains

The TC framework explicitly encompasses five discrete, but mutually reinforcing, skill domains. Transnational competence involves mastery of analytic, emotional, creative/imaginative, communicative, and functional skills. Each skill domain encompasses multiple dimensions.

### Transnational analytic skills

The analytic domain of TC preparation focuses on developing the ability to gather and analyze evidence related to the patient's health rather than on stored knowledge, while recognizing that a knowledge-based approach can be useful for specific and limited purposes [27,48]. In particular, TC education recognizes the necessity to probe beyond ethnicity/culture. As Moustafa Bayoumi observes, "by obsessively focusing on culture, we avoid talking about history, economics and politics" [[77], p.A4]. In short, an exclusively ethnic/cultural observation "obscures the social and structural basis of the need ..." [[37], p.34]. The interweaving of ethnocultural, socio-political, and medical analyses is required for comprehensive assessment of each patient's health-care needs.

To avoid misinterpreting messages and explanations offered by patients in spatial transition, medical students must develop expanded receptors for discerning political and socio-economic determinants of individual health; [78-82,62] that is, they must learn how to perceive health situations through what Mary Duffy refers to as the patient's "global lens" [[36], p.489]. In particular, it is important that medical practitioners elicit and explore the longitudinal dimensions of spatial *transition *given that established as well as recent migrants often are dealing with "unfinished endings" that preceded their arrival in the current locale [[38], p.89, [83]] and continue to shape their lives [84,85]. Physicians possessing transnational analytic skill are able to comprehend and critically appraise the internal and external forces that affect migration health [86,87] by expanding the medical discourse to include linked macro-structural and micro "origins of personal suffering" [[63], p.276] – such as war, [48,88,89,84] manipulations of national and subnational economies by powerful global institutions, [87,90-92,12] foreign policy, [86] powerlessness, [93] persecution, and the type, combination, and frequency of trauma experiences [94].

Transnational analytic skill further involves unraveling existing linkages between migrant health and post-migration constraints and stressors associated with receiving-country reception practices and new developments in the country of origin [95-97]. For instance, a patient's capacity for self-care can be limited by ongoing "cultural and linguistic isolation, fragmentation of the family, deformation of social relationships, chronic absence of adequate support systems, poverty, prejudice, and unemployment" [[98], p.32, [59], [63,99-103]] – all rooted in migration and post-migration experiences. Furthermore, political and family events and conditions in the sending country often continue to affect the mental health and physical well-being of service users who possess transnational ties and identities [104,84].

Another critical transnational analytic skill in migration health is the ability to ascertain the role of ethnocultural and other nonstandard health-related beliefs, values, practices, and paradoxes. The "transnational healing" practices of some contemporary migrants even include return to the country of origin for medical attention and treatment [105,106]. TC education prepares students to assess the role of nonbiomedical considerations in the pre- and post-migration explanatory model and decision-making processes of specific patients and/or families [107-112]. Box 1 presents illustrative TC-preparation components in the analytic domain.

### Box 1

#### Illustrative TC-preparation components: Analytic domain

1. Develop the theoretical base for analyzing the particular socio-economic and political factors that mediate experience and influence health-care delivery for the individual patient.

a. Introduce useful concepts from waste and consumption studies [113,114]

i. notion of a *chain*, with individual decision nodes that tend to be severed from contextualized understanding of shaping and constraining upstream and downstream social forces and power relations that invoke hidden costs.

ii. process of *distancing*; that is, stretching the chain (geographically, culturally, and mentally). Mental distance includes gulfs of information, awareness, and responsibility.

iii. possible applications when analyzing transnational health care

1. recognize the need to move upstream and downstream along the health chain in the effort to uncover specific case-relevant contextual social forces and power relations. Raise consciousness that individual medical care alone cannot be sufficient to sustain practices that will maximize the patient's health potential.

2. recognize that moving upstream and downstream intergenerationally is likely to yield divergent as well as overlapping insights.

b. Connect concepts (including class, identity, power, and distancing) to the ability to discern and analyze critically [64] the distant political/economic/social/environmental contributors to proximate health "variability, vulnerability, and strength" [[33], p.579, [115]]; specifically, the interaction of dislocation and transit experiences (including types, extent, and duration of persecution and trauma) with:

i. different migration decisions and forms of migration – forced, planned and long-term, planned and short-term [2]

ii. structural inequities embedded in conditioning institutions

iii. linked macro and micro, local and global forces

c. Connect concepts (including class, identity, power, and distancing) to the ability to discern post-migration conditions affecting the patient's current health-related beliefs and practices and physical and mental health in the receiving society. Potentially influential post-migration conditions include:

i. social/political experiences and stressors

ii. simultaneous and potentially conflicting home- and host-country expectations and medical treatments

iii. differential access to health-care system and treatment opportunities

iv. altered nutrition practices

v. immigration status

vi. occupational and employment transitions

vii. (il)literacy and education

viii. housing & transportation situation

ix. (lack of) support networks

x. extent, and positive and negative effects, of adaptation [31]

d. Connect concepts to skill in discerning life-style and health consequences of the patient's changing *class profile *- often characterized by radical downward mobility in the case of involuntary (politically dislocated) migrants and upward mobility for voluntary (economic) migrants (accompanied by exposure to new risks and the adoption of detrimental health behaviors)

2. Develop ability to discern the patient's ethnocultural identification(s) and personal (including nonbiomedical) beliefs and practices regarding causes, treatment, and prevention of illness.

3. Develop understanding of how the degree of one's cultural, ethnic, and socio-economic match with the patient influences the therapeutic relationship [115]. Learn to avoid the "cultural blind spot syndrome" where the clinician assumes no distinctive health-care beliefs/practices exist because the patient looks and behaves much the same way as s/he does [116,117,28].

4. Develop ability to utilize analytic techniques transnationally

a. by locating and learning from helpful proximate and reliable current sources

i. ethnic community

ii. ethnic health specialists

iii. intercultural mediators

iv. other care providers (nurses, social workers)

v. internet & telemedicine

vi. published research findings

b. by using general information about population-specific disease incidence/prevalence/outcomes, new and emerging diseases, and antimicrobile resistance [2] and the patient's places of origin and transit, ethnicity, cultural and spiritual practices, previous sources of health care, migration/trauma experiences, economic situation, degree of societal incorporation, and support systems as a starting point for physical/mental-health inquiry, confirmation/ disconfirmation, and recommended therapies/referrals

c. by accessing and assessing information regarding the pharmacological properties of the care recipient's ethnocultural preparations (ethnopharmacology) [48]

d. by eliciting comprehensive patient narratives and explanatory frameworks that move beyond the prevalent "brief and perfunctory social history" [118]

### Transnational emotional skills

Transnational emotional competence includes the ability to express interest in different cultural patterns – language, family life, dietary practices, [119] customs, etc [120] – and the ability to gain and maintain genuine respect for a multiplicity of values, beliefs, traditions, experiences, challenges, preferred communication styles, and feelings of satisfaction and emotional distress stemming from social circumstances [63,34]. Among medical students preparing for encounters with patients of multiple nationalities and diverse identities, the emotional skill domain is developed through interest in interacting with ethnically, culturally, and economically diverse patients. The application of transnational emotional skills requires a "willingness to try" to decipher the patient's thoughts and perspectives [[32], p.1058, [121]] – including his/her beliefs regarding the mediating effect of "luck, chance, randomness and personal destiny" on healthy lifestyles [[122], p.679] – and to respond empathically with an appropriate emotion of one's own [123].

In the migrant-health interface, it is particularly important that care providers learn to respect rather than dismiss lay expertise [72,37] as well as nonbiomedical practices that affect acceptance of and compliance with treatment protocols and, therefore, influence outcomes [124-127,112]. Emotionally skillful participants also appreciate that every clinical encounter is a multidimensional interaction among the cultures of the patient, the physician, the support professional(s), and the health-care contexts/systems that surround them [128,107,48,62].

The emotional-competence domain of a TC education further emphasizes appreciation for the ability of people in spatial transition to regain emotional strength and functional capacity following adversity [129]. Many "refugee patients and their families bring to health consultation stories of incredible human resilience in the most extreme circumstances" [[130], p.27, [110]]. Studies show that a sense of personal, family, and/or group efficacy constitutes a powerful determinant of the adoption and maintenance of health-promoting actions and is associated with a host of health-enhancement and illness-prevention outcomes [131-133,58]. Under the vulnerable and stressful environmental conditions that migrants face as the result of formidable language and cultural constraints, discrimination, the threat of long-term unemployment, and/or lack of social support, clinician appreciation for patient/family health-care assets, capabilities, and responsibilities reinforces individual and collective perceptions of transnational efficacy and strengthens confidence, perseverance, and power to sustain new and demanding psychological and physiological health-enhancing behaviors [132]. Capable self/family illness management is particularly valuable in treating many chronic diseases [134]. Among medical students, emotional competence also involves self-monitoring and reflection; that is, life-long openness to critical self-appraisal, to learning in place of stereotyping, [135] and to promoting emotional growth [136]. Box 2 presents illustrative TC-preparation components in the emotional-skill domain.

### Box 2

#### Illustrative TC-preparation components: Emotional domain

1. Develop abilities to realize health-care insights through transnational empathy, to be effective at deciphering the patient's perspective, to see and take seriously problems as the ethnoculturally discordant patient experiences them, [137,138] and to deliver an appropriate and reassuring emotional response.

2. Develop ability to reinforce/restore efficacy among ethnically and socio-economically diverse patients

a. by demonstrating appreciation for emotional resources (resilience) and achievements in surviving and overcoming dislocation and migration challenges and/or disparities in treatment [115,139]

b. by validating and protecting family-care and self-care practices that facilitate adaptation and well-being [140]

c. by identifying what patients and their support network can do for themselves with some initial outside help [141]

d. by conveying an optimistic outlook on prospects that the patient's health-care needs can be met [142,143]

3. Develop ability to show respect for (acknowledge and validate) the patient's ethnocultural and other nonbiomedical health beliefs and practices – to treat them as distinctive rather than inferior or deviant.

4. Develop ability to motivate health improvements through transnational sociophysiologic feedback [137]. This ability is important because many patients look for help in dealing with the emotional aspects of chronic or other illness and are shocked when clinicians approach their case only in terms of technical efficiency [144].

### Transnational creative/imaginative skills

The freeing up of imaginative capacities is a powerful force for positive health outcomes in the transnational medical encounter [145]. A key creative skill for medical students preparing for migrant-health care is the ability to initiate fruitful new connections among distant and proximate parts of the patient's experience [146]. Skillful transnational clinicians are "creative synthesizers" [[147], p.17, [148]] who value collaboration with, and are able to inspire, participants of diverse identities (patients, family members, and transcultural mediators) in the co-design and nurturing of innovative and contextually appropriate health-action plans [149].

A substantial proportion of all health care is provided "outside the perimeter of the formal health care system" [[150], p.251]. In the migrant-health arena, innovative approaches to managing demands for medical treatment and wellness promotion include complementary integrations of biomedical and ethnocultural explanatory frameworks and health-related practices [107,124,109,151-153,108,96] and incorporate multilevel linkages of individual, socio-political, and ecological considerations [154,155]. In the interest of preparing creative medical practitioners, TC education emphasizes flexibility and adaptability when confronted with unique and unfamiliar situations [37].

Imagination "makes empathy possible" by lending "credence to alternative realities" [[146], p.3]. Medical practitioners must be prepared to relate physical and emotional experiences and perceptions that shaped the decision to leave the country of origin, as well as those that arise during migration and resettlement processes, both to approaches that effectively address the patient's current health-promotion needs [152,155] and to promising social changes and policy alternatives [63]. Box 3 presents illustrative TC-preparation components in the creative/innovative domain.

### Box 3

#### Illustrative TC-preparation components: Creative domain

1. Ability to account for the patient's current place-specific environment (housing, social dis/organization, transportation, employment, etc.) in the tailored health-action plan.

2. Ability to forge synergetic and congruent linkages between what the patient believes and what the clinician believes [28].

3. Ability to co-create a health plan based on shared transnational synthesis – a complementary combination of biomedical and personal (ethnocultural/mixed-cultural) beliefs/practices that is neither clinically, culturally, nor economically contraindicated [115,156,81].

4. Ability to activate and incorporate the patient's own ideas, suggestions, resources, and ingenuity into the mutually agreed-upon health plan.

5. Ability to account for the ethnoculturally discordant patient's unique life context (physical and emotional experiences and institutional forces) in the tailored health-action plan.

6. Ability to construct a tailored health-promotion action plan that includes societal reinforcement for linked physical/mental-health interventions [115].

### Transnational communicative facility

Effective provider-patient communication is widely perceived as "a core competency in the health care profession" [[59], p.27, [58,48]]. While personal linguistic fluency in the patient's first language is an immense behavioral asset, [157-161,48] achieving it is impractical in transnational health-care situations involving multiple first languages [32]. In New York City, for instance, patients might speak one of 150 different languages [162] Thus, TC education emphasizes skill in using an interpreter, the importance of employing trained medical interpreters, [163-166,153,158,160,111,157,48] and host-language preparation and communication training for patients [134].

Transnationally skillful actors also develop proficiency in nonverbal-communicative behavior. In medical encounters, "nonverbal communication skills ... are as important as verbal skills, if not more so" [[167], p.2445]. In transnational medical interactions, interview pace, speech-simplification strategies, and the use of "continuers" ensure that participants are not rushed, prematurely interrupted, ignored, or incompletely understood [168-170,164]. In addition, communication-recovery skills, such as humor, apology, and admission that one does not know everything, "reinforce confidence as well as competence because, when it is known that there is something to fall back on, one is less likely to avoid interactions that may prove difficult" [[96], p.245, [135]].

The capacity to engage in meaningful dialogue and to facilitate mutual self-disclosure via questioning is particularly important in transnational health-care situations characterized by vast social distance [168]. Similarly, a prerequisite for negotiating appropriate treatment plans and commitment to agreements is that participants – especially migrant patients – are comfortable expressing serious doubts and constructive challenges [168,63,58]. Box 4 presents illustrative TC-preparation components in the communicative-skill domain.

### Box 4

#### Illustrative TC-preparation components: Communicative domain

1. Ability to select the most helpful interpreter for each patient's specific cultural, linguistic, and social context

2. Ability to use best practices associated with the participation of interpreters in clinical consultations [156]

3. Proficiency in patient-appropriate non-verbal communication

4. Proficiency in active listening and taking the patient seriously [138]

5. Ability to use speech-simplification strategies

6. Communication-recovery skills

7. Ability to facilitate mutual self-disclosure [33]

8. Ability to convey health-care options and recommendations across language and cultural divides

9. Ability to elicit patient's questions and concerns

10. Ability to elicit patient's doubts and disagreements

### Transnational functional adroitness

Functional competence involves the interpersonal as well as technical ability to accomplish tasks and achieve objectives. In transnational medical encounters, the functional skills of both patients and clinicians affect illness management and wellness promotion [171,172]. In migrant-health-care consultations, effective functional interventions take into account both the individual's condition and the social context affecting health behavior [155].

Skill in establishing positive interpersonal relations is particularly valuable for the functional domain of migrant-health care. Keys to success in building fruitful transnational relationships include demonstrating genuine and sustained personal as well as professional interest in the care recipient as an individual, commitment to the patient's cognitive and instrumental needs, [137] and support for his/her social inclusion [36]. TC preparation emphasizes that, in the case of migrants who lack voice in the socio-political context they find themselves in, concern for patient well-being can be demonstrated by actions that address factors responsible for personal suffering [63]. Valuable relationship-building TC-provider interventions include helping with transportation to medical appointments, facilitating access to traditional healers, medicine, and nutrition, promoting ties to community support networks, identifying and enhancing the development of "new roles that provide a sense of meaning and structure to daily life," [[173], p.294] and assisting with host-country language training, further education and credential (re-)certification, employment, and the maintenance of (children's) healthy practices.

The functional dimension of transnational competence also is promoted by establishing clinician/patient partnerships, or "therapeutic alliances." [135] In the transnational therapeutic alliance, "the process of negotiation between practitioner and patient involves developing courses of action that are consistent with the patient's values and goals and that also satisfy the physician's values and goals ..." [[168], p.13, [33]]. For many migrants, transculturally sustainable agreements must include involvement by (extended) family members and/or migrant-community support networks [111,150].

In the interest of equitable health opportunities for migrant patients, transnational functional adroitness necessitates advocacy competence; that is, recommendations/actions that will generate upstream and downstream changes in domestic and international economic, social, institutional, and policy conditions that produce the systemic disparities that constrain individual health and preclude the realization of health gains [23,174-177,92,48,76,94]. It is likely to be particularly rewarding for functional skill development to focus students' advocacy attention on local "hot spots" where migrants tend to congregate. In this part of functional TC preparation, medical students can be guided to develop specific interventions that address context- and site-specific conditions that are conducive to elevated risk-taking behavior [47]. Box 5 presents illustrative TC-preparation components in the functional domain.

### Box 5

#### Illustrative TC-preparation components: Functional domain

1. Ability to establish and maintain meaningful transnational inter-personal relations [178].

2. Ability to relate to ethnoculturally and socio-economically discordant patients in a way that builds mutual trust

a. by showing that one genuinely is interested in, cares about, and is committed to helping with the patient's current situation and quality of life (beyond physical health) [179,138]

b. actions are regarded as appropriate and useful

c. conflicts are resolved to mutual satisfaction

3. Ability to apply relevant insights from the other four TC domains.

4. Ability to integrate evidence-based insights regarding the influence of ethnocultural practices and disease predispositions, class, access, migration, and trauma into patient-specific health-status hypotheses and effective health-care responses.

5. Ability to engage the patient (and/or his/her family) in making joint health/illness assessments and in developing/modifying health-promotion plans [81,180]. At times, this process requires the ability to overcome structural constraints that limit the amount of time available for consultations with patients [181].

6. Advocacy and referral skills I. Ability to build and activate host-society and migrant-community resources that are likely to enhance the patient's health situation by mitigating the site-specific environmental constraints they confront.

7. Advocacy and referral skills II. Ability to build and activate societal resources that are likely to enhance the patient's health situation by mitigating the socio-economic inequities, power differentials, exclusion policies, and other institutionalized constraints they confront.

### TC pedagogical approaches

Along with introductions to challenging new material and helpful insights regarding contemporary medical practice, it is critical that future physicians be "taught in a way that works better" [182]. For maximum effect, the core elements of a TC education need to be longitudinally woven into required pre-clinical and clinical education through instructional approaches that encompass lectures, small-group discussions that include reference to the consequences of patient stereotyping, analysis of written and videotaped case studies, constant reference to clinical applications, interaction with community leaders, training in interviewing skills, as well as experiential approaches such as role plays, [27] encounters with simulated patients, overseas immersion, [8,36] involvement in community service-learning projects, and carefully designed clinical clerkships. The didactic components of the longitudinal and integrated TC approach would establish the need for adaptable skills in the contemporary context of globalization and health, would build a comprehensive foundation of five skill domains, would highlight the special value of experiential learning and reflective practice when attending to migrants, and would emphasize the centrality of collaborative efforts to promote social justice in health care through multi-dimensionally sensitive and individual-patient responsive transnational medical encounters. Resources from the humanities (e.g., art, literature, autobiographical accounts of migrant-patient experiences) can be especially useful in the initial effort to awaken the student's imagination [146] and to convey TC concepts that are inherently important in caring for migrant patients [183]. To facilitate stakeholder buy-in, the instructional and experiential dimensions of TC medical education also require attention to faculty-selection criteria, resources for faculty development in skill-deficit domains and in unfamiliar pedagogical approaches, and institutional as well as external support for materials development, contributions by specialists, assessment exercises, and logistical arrangements. Medical schools and teaching hospitals also will need to reinforce or establish linkages with often fragmented migrant-community associations and with community-health advocates.

In contrast to educational methods that center on mastery of ethnic patterns of disease or lists of cultural characteristics, the predominantly inductive TC approach focuses on the patient as the starting point for discovery and avoiding mistakes [27,184]. When health-care providers work with diverse service seekers, skill development occurs through "bottom-up" information and evidence gathering that places primary emphasis on contextual insights derived from proximate and current sources – the patient himself/herself and family, friends, and/or community members [185,48]. In light of the existence of national subcultures and the presence of intracultural (and changing) variations that occur due to "age, gender, income, education, acculturation, individual differences, and multiple other factors," general epidemiological evidence about the patient's country and its endemic diseases, ethnic group, or religious affiliation needs to be "regarded as having some bearing but requires further validation to be considered immediately useful" [[185], p.251–252, [186,96,27,33]]. As Melanie Tervalon and Jann Murray-Garcia point out, "only the patient is uniquely qualified to help the physician understand the intersection of race, ethnicity, religion, class, and so on in forming his (the patient's) identity and to clarify the relevance and impact of this intersection on the present illness or wellness experience"; that is, "how little or how much culture has to do with that particular clinical encounter" [[135], p.121].

For TC preparation, therefore, skill development is expected to be especially robust during the student's clinical-clerkship experiences. In a TC-informed medical education, exposure to transnational medical encounters would constitute an integral part of all clinical clerkships. Clerkships that involve migrant patients present students with a variety of stimulating medical challenges framed by diverse cultural perspectives and social backgrounds [65] and, simultaneously, provide problem-solving opportunities for students to articulate helpful recommendations and rewarding interventions. When designing each TC clerkship experience, faculty would arrange for students to work closely with patients and family members from distinct and diverse cultural, ethnic, subcultural, generational, and socio-economic backgrounds. Gerrish, Husband, and Mackenzie warn that "a *de facto *emphasis on cultural competence, with a resultant neglect of intercultural competence, must be resisted" [37,134,135]]. Thus, TC clinical placements and preceptor-supervised encounters with patients [121] would avoid focusing on a single local population. Clinical assignments also should proceed to levels of increasing complexity and be linked to reflective seminars that involve sharing and group discussion of case-specific and transnational issue-related insights gained from interviews with multiple patients of diverse backgrounds and from students' health-promotion and social-context (advocacy) recommendations. For educators working at institutions in the few rural areas or population centers that remain relatively untouched by migration, the experiential component might need to supplement a relatively homogeneous patient base through student participation in out-of-country immersion programs, cooperative arrangements with urban medical schools, and/or videoconferencing [187].

TC clerkships would emphasize the validation and promotion of factors that facilitate health recovery/maintenance, transnational adaptation, and survival. When working with ethnoculturally discordant patients, "the *ability to identify assets *in a family beset by overwhelming liabilities" as well as vulnerabilities "often produces the turning point toward successful interventions" [[188], p.269]. The bases for resilience vary among patients and are subject to change over time [189]. Possibilities for students to explore include: hopeful vision for the future; religious faith; self-reliance; personal history of overcoming adversity; roots; finding meaning/purpose in life; [189] and community mutual assistance and support [190]. In TC clerkships, students would learn that unduly pathologizing the migrant's experience [118] exaggerates deficiencies, risks fostering dependency, [191] and "removes the matter from the political and social context that produced ... [the] anguish and loss" [139]. TC clinical education also aims to provide the future physician with a toolbox of ways of reinforcing and expanding resilience (especially preparing patients to take responsibility for self care and problem solving in a confident manner, which often involves family and nonbiomedical supplements and addressing resource needs), reversing devaluation and disempowerment by providing opportunities for patients to demonstrate and develop role competence and increased control over their life both in and beyond health-care situations, [192] and enabling migrants to resist the adoption of health-adverse behaviors practiced by members of the receiving society [193,194]. Furthermore, TC clerkships would demonstrate that the extra time spent on caring behavior (estimated at 5–7 minutes per encounter until the caring relationship is established) results in multiple benefits for both practitioner and patient [137,181]. Increasingly, managed-care providers recognize that providing such quality attention more effectively contains health-care costs than does limiting services [115].

A central component of inductive TC pedagogy and the TC clinical clerkship is a "mini ethnography" of health, illness, and migration/adaptation experiences [80,195]. In the transnational medical encounter, the patient's narrative of lived experience – including the migrant's stressful social and environmental situations, network of transnational social relations, and emerging identities [95] – is particularly valuable [145,96]. Genogram construction [178] constitutes another illuminating tool that can be built into the ethnographic interview. The ethnographic-learning experience should include observations in the patient's social territory; critical reflection on the medical impact of power relations, institutionalized constraints, and patient/family strengths; opportunities for the patient to comment upon the student's initial findings; preceptor feedback regarding the strengths and limitations of each student's interviews; and facilitated discussions with faculty in small-group settings of the students' findings as well as possible hidden social, economic, legal, and cultural contributors. The ethnographic approach reduces prospects that decisions will be based on stereotypic oversimplifications and/or insufficient information [196,48,128,112] and helps medical practitioners avoid the overgeneralized tendency to perceive and treat migrants as traumatized victims [95].

Ethnographic interviewing also needs to be linked to skills in documenting how patient/family perspectives and insights that bear upon the patient's physical and mental health as well as his/her current social, economic, and legal circumstances will be addressed in the recommended health plan. In her case study of Lia Lee's treatment by U.S. doctors, Anne Fadiman reports that Lia's medical chart "grew longer and longer, until it contained more than 400,000 words. ... [Yet] not a single one dealt with the Lees' perception of their daughter's illness" [[81], p.259].

The TC approach involves explicit expectations that students act as the patient's advocate by forging partnerships with community organizations and advocacy groups and by making social-context recommendations that address both short- and long-term challenges to health [180,197]. Preceptors would be expected to provide feedback to students about documented results that arise from their recommendations. TC's advocacy emphasis further suggests the value of integrating community-based [156,64,36] experiential or service learning into the medical student's education [198].

Assessments of TC-learning outcomes would include student course and clerkship evaluations, student self-evaluations and instructor appraisals of pre- and post-classroom learning (e.g., the student's ability to explain why the unique migration history of a refugee from Afghanistan is important for the patient's health care [199]) and humanistic-values enhancement, review of randomly videotaped/audiorecorded encounters with patients of diverse backgrounds, [27] preceptor evaluation of each student's applications of the five TC skills (e.g., the ability to delineate and document a comprehensive plan of action that connects the patient's socio-cultural background, perspectives, and context with his/her current health challenges and promising medical and nonmedical responses), and TC-relevant OSCEs [27,184]. TC skill assessment would be incomplete without eliciting and incorporating *patient reflections *on the interview process, the accuracy of insights reported in the mini-ethnography, the efficacy of the student's proposed and initiated actions in terms of health-promoting interventions and personal health outcomes, [27] and the attending student's overall TC strengths and deficits.

### Funding

Successful implementation of a TC educational initiative requires additional resources. The training and employment of medical interpreters, the conduct of ethnographic interviews, the professional development of medical-school faculty who are qualified to offer TC-informed courses and to supervise TC-centered clerkships, and the construction and execution of systematic evaluation studies constitute critical components of the educational framework presented here that will be well-served by supplemental external funding. This is particularly the case for resource-scarce universities in Southern countries. In addition to internal reallocations, a variety of national and international funding sources can be mobilized for program support. Ideally, the World Health Organization would assume responsibility for driving, and coordinating funding for, the TC initiative. The faculty-development and evaluation components also would be promoted by national government incentive programs carried forward in partnership with higher-education institutions, including the United Nations University. Foundations and associations of medical professionals could usefully contribute to the global TC educational initiative, with some programs specifically devoted to the preparation of professional medical interpreters, transnational navigators, and patient advocates along with TC training for migrants. Grounding in TC, along with receptivity to continued mutual South-North learning, could be fruitfully incorporated into the Bill and Melinda Gates Foundation-supported "E-learning Certification Programme in Global Health" initiated through Oxford University [200] and into the post-graduate educational programs offered by the Department of Global Health at The University of Washington that will be launched in 2006 thanks to another Gates Foundation grant.

## Conclusion

As the diversity of patient populations continues to expand in both North and South, it is time for a proactive and mobility-relevant redirection of medical education on a global scale. In some cases, adopting the TC framework requires fundamental shifts in orientation and approach. Other medical schools are positioned to reinforce skills already covered (e.g., ethnographic interviewing, working with intercultural mediators) within the context of TC's encompassing and globally relevant framework. The advantages of TC-inspired redirection of medical education are manifold. TC preparation (1) provides an integrated and comprehensive set of practical and contemporary medical-consultation skills of value in an age of population mobility; (2) accepts that acquired mastery of the "multiplicity of cultures that comprise the patient populations of today" [[185], p.250] is neither feasible nor necessary for quality care and cost containment; instead, the TC approach focuses on discerning each patient's multiple and complex (rather than single-source) identities and distinctive health perspectives and personal needs in ways that build trust, confidence, and humility; (3) places the physical- and mental-health consequences of economic disparities and underlying global/local structural contributors front and center; (4) aims to equip both service users and service providers with parallel skills [75]; (5) addresses both the quality of patient care and social constraints on migrant health; and (6) applies to and promises to resonate well with clinicians in all countries who work with ethnoculturally and socioeconomically discordant patients. Consequently, a TC education would equip learners for global and not just local practice – an important qualification given the scope of contemporary population and professional mixing.

In our mobility-upheaval era, transnational-competence preparation offers a promising avenue for providing clinicians and other public-health professionals with the full complement of interpersonal skills needed to be effective care providers in the global North and the global South. Exploratory research suggests that TC skills can improve health-care outcomes in ethnoculturally discordant medical encounters, [29,97] although confirmation requires more elaborate and comparative investigations. Given that few medical schools have embarked on pilot TC programs to date, [201] that a full-blown TC curriculum would involve demanding expectations of currently stretched students and faculty, and that compelling evaluation results require additional outcome-based research studies, controls, and time, substantiating claims for the efficacy of TC education remains a future project. However, as the value of preparation in generic TC skills is further demonstrated through student assessment, modeling by clinician mentors, mistake avoidance, [184] patient satisfaction, quality assurance, and reduced health disparities, the future physician's intrinsic human and professional motivation [202] to interact ever more effectively on behalf of ethnoculturally and socioeconomically unfamiliar and disadvantaged patients will provide the foundation for, and facilitate openness to, the development of personal transnational competence in migrant-health care.

## Competing interests

The author declares that he has no competing interests.
